# Advances in understanding bat infection dynamics across biological scales

**DOI:** 10.1098/rspb.2023.2823

**Published:** 2024-03-06

**Authors:** Cecilia A. Sánchez, Kendra L. Phelps, Hannah K. Frank, Marike Geldenhuys, Megan E. Griffiths, Devin N. Jones, Gwenddolen Kettenburg, Tamika J. Lunn, Kelsey R. Moreno, Marinda Mortlock, Amanda Vicente-Santos, Luis R. Víquez-R, Rebekah C. Kading, Wanda Markotter, DeeAnn M. Reeder, Kevin J. Olival

**Affiliations:** ^1^ EcoHealth Alliance, New York, NY 10018, USA; ^2^ Department of Ecology & Evolutionary Biology, Tulane University, New Orleans, LA 70118, USA; ^3^ Centre for Viral Zoonoses, Department of Medical Virology, University of Pretoria, Pretoria, South Africa; ^4^ MRC-University of Glasgow Centre for Virus Research, Glasgow, UK; ^5^ Department of Microbiology & Cell Biology, Montana State University, Bozeman, MT 59717, USA; ^6^ Department of Ecology and Evolution, University of Chicago, Chicago, IL 60637, USA; ^7^ Odum School of Ecology, University of Georgia, Athens, GA 30602, USA; ^8^ Center for the Ecology of Infectious Diseases, University of Georgia, Athens, GA 30602, USA; ^9^ Department of Psychology, Saint Xavier University, Chicago, IL 60655, USA; ^10^ School of Biological Sciences, University of Oklahoma, Norman, OK 73019, USA; ^11^ Department of Biology, Bucknell University, Lewisburg, PA 17837, USA; ^12^ Department of Microbiology, Immunology and Pathology, Center for Vector-borne and Infectious Diseases, Colorado State University, Fort Collins, CO 80523, USA

**Keywords:** biomarkers, Chiroptera, disease ecology, health, stress, physiology

## Abstract

Over the past two decades, research on bat-associated microbes such as viruses, bacteria and fungi has dramatically increased. Here, we synthesize themes from a conference symposium focused on advances in the research of bats and their microbes, including physiological, immunological, ecological and epidemiological research that has improved our understanding of bat infection dynamics at multiple biological scales. We first present metrics for measuring individual bat responses to infection and challenges associated with using these metrics. We next discuss infection dynamics within bat populations of the same species, before introducing complexities that arise in multi-species communities of bats, humans and/or livestock. Finally, we outline critical gaps and opportunities for future interdisciplinary work on topics involving bats and their microbes.

## 1. Introduction

Studies of bat-associated microbes (i.e. microorganisms detected in or isolated from bats) date back to rabies virus investigations in the early 1900s [1]. In the past two decades, following the emergence of Severe Acute Respiratory Syndrome (SARS) coronavirus (CoV) in 2003 and SARS-CoV-2 in 2019, there has been a dramatic increase in research on bat-associated microbes, including viruses, bacteria, haemosporidians and fungi [2–5]. These microbes may or may not cause disease in bats, and thus we broadly use the term ‘microbes’ rather than ‘pathogens’ throughout this paper to acknowledge that detecting microorganisms in bats is distinct from the process of determining pathogenicity [6]. Research has moved far beyond simple microbe detection in bat hosts and includes cutting-edge investigations into infection dynamics at individual, population and community scales, and One Health approaches to integrate bat ecology and health [7–11].

**Figure 1.**
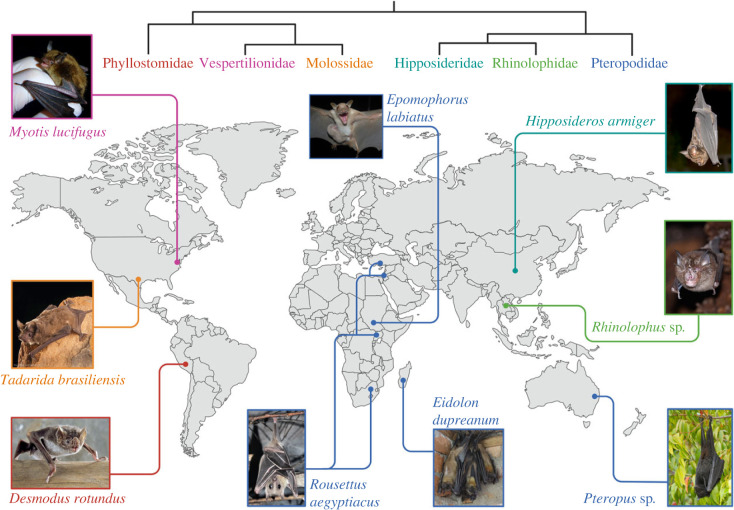
Map illustrating the geographical and taxonomic diversity of bat species highlighted in case studies throughout the main text, with approximate study location and photo. Species names are coloured according to bat family, with a simplified phylogeny showing relationships between families. See electronic supplementary material for photo permissions.

As part of the joint 50th North American Symposium for Bat Research and 19th International Bat Research Conference, we organized a symposium focused on advances in the research of bats and their microbes (electronic supplementary material, table S1). We invited early-career scientists to present on physiological, immunological, ecological and epidemiological investigations that have improved our understanding of bat health and infection dynamics. Building on topics discussed by our presenters, here, we review recent bat infection research at the individual, population and community scales. We first present novel approaches and metrics for measuring individual bat responses to infection and challenges associated with assessing consequences of infection. We next discuss infection dynamics within bat populations of the same species, before introducing complexities that arise in multi-species communities, including humans or livestock. Throughout, we highlight case studies from a diverse set of bat species (figure 1). We conclude by summarizing critical gaps and opportunities for future interdisciplinary work on health topics involving bats and their microbes.

## 2. Research at the individual scale: metrics for assessing bat responses to infection

A prevailing narrative in infectious disease research is that bats do not get ‘sick’ when infected with viruses or other microbes (with some exceptions [12]). Experimental challenges on individual bats and bat cell line infection studies have reinforced this narrative, suggesting bats may be more tolerant of viral infection than other mammals [13–15], especially for microbes for which they are the putative reservoir host. However, other studies suggest bats sometimes develop disease due to microbial infection (e.g. morbidity and mass mortality events caused by viruses, fungi and bacteria [16–18]). We submit that researchers must employ a broader set of metrics and technologies to build a more complete picture of bat responses to infection.

### (a) Physiological responses to infection

While acute responses to microbial infections may have minimal consequences for physiological status, cumulative and/or interactive effects of co-occurring or repeat infections can precipitate a cascade of detrimental physiological responses [19]. A comprehensive strategy to assess bat responses to infection should include complementary ‘snapshot’ indicators that show individual short-term reactions as well as downstream metrics that reflect prolonged physiological responses.

A reliable tool for examining the relationship between physiological status and infection status in bats is the measurement of glucocorticoid (GC) hormones. GCs (i.e. cortisol and corticosterone) are critical in regulating physiological processes (e.g. metabolism, reproduction, immunity). While short-term increases in GCs are beneficial for survival, prolonged elevated levels of GCs can downregulate immunological functioning, potentially increasing vulnerability to infection and transmission risk to other species [20,21]. Minimally invasive (e.g. blood, < 5 µl) and non-invasive (e.g. faeces, urine and fur) methods to quantify GCs are increasingly available [22,23].

Body condition can serve as a downstream indicator of the consequences of infection [24], with studies identifying associations between decreased body condition and infection status [25–27]. However, significant variation in morphology among bat species means a one-size-fits-all measure of body condition may not exist. The most widely used body condition indices (BCI) are the ratio index (body mass/forearm length) and the residual index (residuals of body mass-forearm length regression), which attempt to provide size corrections for body mass [28]. For temperate insectivorous bat species, body mass alone has been suggested as a more effective measure of body condition [24]. Regardless of the index used, it is worth noting that short-term factors affecting mass (e.g. pregnancy, food consumption, waste elimination) can alter BCI values and interpretation.

With a small amount of whole blood (< 100 µl), researchers can assess a bat's physiological status via blood chemistry parameters. For instance, handheld point-of-care blood analysers (e.g. i-STAT) were used to demonstrate that little brown bats (*Myotis lucifugus*) infected by *Pseudogymnoascus destructans* (the aetiological agent of white-nose syndrome (WNS) [29]) had depleted electrolyte levels and exhibited respiratory acidosis [30,31]. In Ethiopian epauletted fruit bats (*Epomophorus labiatus*), haematological and electrolyte values varied by infection intensity with the malarial parasite *Hepatocystis* [32,33]. Although blood chemistry analysis is promising, it is important to establish reference ranges to serve as a baseline against which measurements from infected individuals can be compared [34–36].

Blood smears, easily prepared in the field from < 5 µl of blood, are a tool by which to characterize leucocyte (white blood cell) profiles that provide a window into the immune status of individual bats [37]. Because leucocytes are energetically costly to produce and maintain, a high leucocyte count can indicate a robust cellular or inflammatory response to acute infection [38]. Neutrophils and lymphocytes are associated with the innate and adaptive immune responses, respectively [39]; therefore, the ratio of neutrophils to lymphocytes is used to measure the relative investment on each arm of the immune response and as an indication of acute infection or chronic stress [40]. As with other physiological metrics, we lack an understanding of baseline values and the interpretation of ‘abnormal’ leucocyte profiles in bats. Promising lines of work include the validation of markers for more detailed classification and study of bat lymphocyte types (e.g. T-cell subsets, B-cells, natural killer cells [41]), and studies of B- and T-cell receptors [42], the characterization of which will greatly improve our understanding of bat infection responses.

Transcriptomic approaches, in which a snapshot of expressed genes is sequenced and identified, have proven invaluable in understanding the severity of metabolic and immune consequences of infection for bats [15,43,44]. Additionally, the blood proteome contains proteins secreted from blood cells and organs, including those involved in host response to infection and immune biomarkers, and innovative proteomic tools show potential in characterizing bat immune systems and their responses to microbial infections [45]. Complemented by recent advances in genomics [46], ‘-omics’ approaches stand to further our ability to explore mechanisms by which bats interact with microbes and consequences for bat physiological status.

### (b) Behavioural responses to infection

Sickness behaviours are largely consistent across vertebrate species and include decreased movement, food consumption and social interactions [47,48]. However, few studies have focused on how bats alter their behaviour during illness. Several species (*M. lucifugus*; common vampire bat (*Desmodus rotundus*); Egyptian rousette (*Rousettus aegyptiacus*)) reduce overall activity levels when experiencing immune challenges (e.g. lipopolysaccharide injections) or microbial infections [49–51]. Additional behavioural changes include social isolation, temporary cessation of foraging flights, and reduced grooming, production of contact calls and food intake [49–54]. Given the diversity of bat species, data from only three species is insufficient to fully describe how bats alter their behaviours when infected.

Understanding behavioural responses to infection is also important for designing and interpreting microbe surveillance studies, given that infected individuals may be underrepresented in sampling due to a reduction or cessation of foraging [51]. Most knowledge of behavioural changes comes from work with captive bat colonies, allowing for continual monitoring of behaviours of interest. However, two studies tracked free-ranging bats [51,52], demonstrating the feasibility of observing behavioural changes in free-ranging bats in the context of infection. Ongoing technological advances will continue to expand opportunities for monitoring previously inaccessible bats. For instance, smaller on-animal trackers and batteries will enable movement studies for smaller species [55]. Automated video analysis tools, combined with thermal imaging cameras, will enable monitoring of departures from typical behavioural patterns in high bat density environments [56–58]. Identifying disruptions to typical patterns will require robust long-term baseline behavioural data for multiple species. Collaborations between disease researchers and those conducting long-term behavioural studies [59] would be especially valuable in this context; individual-scale, longitudinal infection data could be added to behavioural studies to understand changes linked with infection status.

### (c) The role of bat microbiomes in regulating infection

Much research has demonstrated the importance of host-associated microbiomes, particularly in the gastrointestinal tract (GIT), in influencing host immune function [60]. However, the extent to which the GIT microbiome affects the ability of bats to maintain or mitigate infections remains largely unknown [61]. Destabilization of gut and other symbiotic microbial communities (i.e. dysbiosis) can negatively affect an individual's immune status [62]. Experimental studies have shown that GIT microbiota transplanted from great roundleaf bats (*Hipposideros armiger*) into antibiotic-treated mice altered immune cell levels and conferred greater resistance and survival to H1N1 influenza infection compared with control groups, indicating the GIT microbiome can interact with and change the host immune system [63]. Additionally, lipopolysaccharide injections in *R. aegyptiacus* induced significant and rapid (24–48 h) changes to the composition and diversity of gut microbial communities [64].

Many questions regarding the relationship between GIT microbial communities and bat health remain, including: how do GIT microbes interact with host immune function to maintain, prevent or clear infections? To what extent do GIT microbial communities differ and influence responses to infection relative to other mammalian species, especially given rapid gut transit times in bats? How do microbial communities change naturally over time or with viral, bacterial or other active infections? Repeat sampling of individual bats will aid our ability to answer these questions and identify how dysbiosis presents in bats. Studies of bat microbial communities paired with whole-genome sequencing, transcriptomics, metabolomics and viral screening approaches will provide a holistic picture of tolerance and resistance mechanisms.

## 3. Research at the population scale: elucidating patterns of infection dynamics

The often-gregarious nature of bats allows researchers to examine links between population demographics (e.g. age composition, density) and population measures of infection such as prevalence and seroprevalence. Different sampling methods (e.g. cross-sectional versus longitudinal) can provide an understanding of infection at a single time point or across time scales. Data collected in the field can be used to develop and validate mechanistic models to understand how viruses are maintained in bat populations [65]. Relatedly, model-guided fieldwork approaches are useful to focus data collection on the key drivers of infection dynamics, and to maximize the power of inference during data analyses [66].

### (a) Linking population characteristics to infection dynamics

Population-scale demographics can significantly influence infection dynamics. Seasonal reproductive cycles are common across bat species and are thought to mediate population-scale infection dynamics [67–69]. During gestation, immune function is biased towards anti-inflammatory responses that are important for a successful pregnancy but can increase virus susceptibility within females [70]. These shifts are modulated by hormonal changes that trigger an anti-inflammatory polarization of immune cells [70]. Bats, which rely particularly on inflammatory innate and cellular responses for heightened viral suppression and regulation of latent infections, are expected to be heavily influenced by a gestation-induced anti-inflammatory polarization [71]. Immunological shifts could explain seasonal and sex-specific biases commonly observed in bat antibody seroprevalence [72–74], and seasonal patterns in shedding and spillover [75]. The importance of reproduction in infection dynamics remains to be investigated in a mechanistic fashion, partly due to challenges in sampling sufficient individuals per demographic or reproductive cohort for meaningful analyses.

Seasonal dynamics relating to the influx of susceptible juveniles have been studied in detail and have been associated with increased infection prevalence in populations. For example, pulses of Marburg virus infection have been noted in older juvenile *R. aegyptiacus* in Uganda, co-occurring with synchronous bi-annual birthing cycles [76]. The combined effects of waning maternal antibodies and immunologically naive bats roosting beneath adult bats contribute to the circulation of Marburg virus in this reservoir host [76]. Similar viral dynamics have been reported for diverse henipavirus-related viruses among *R. aegyptiacus* [67], and ‘amplification’ cycles for coronaviruses in multiple other species [25,68,77,78].

Roosting preferences relating to habitat type and aggregation patterns often correlate with infection dynamics [79]. Cave-roosting species typically exhibit higher rates of infection and a greater diversity of viruses than non-cave-roosting species [80]. For tree-roosting species, within-roost aggregation structures can mediate infection dynamics. For example, sparsely distributed tree stands can promote high within-tree bat densities due to limited tree availability, further promoting transmission and generation of more explosive epidemics upon virus introduction [81]. Not all species within a genus roost in the same densities. For instance, Asian *Rhinolophus* species linked to SARS-related coronaviruses (sarbecoviruses) roost in higher densities and with more species than European and African *Rhinolophus* sarbecovirus hosts [82], increasing risk of viral recombination and adaptation to new hosts.

### (b) Sampling strategies to infer population-scale infection dynamics

Biosurveillance among bat populations has traditionally been performed opportunistically and as cross-sectional studies [83]. One-time cross-sectional sampling can provide an excellent overview of microbe presence and diversity within and across host species, as well as insights into tissue tropism and routes of excretion [84,85]. Opportunistic sampling across diverse species has also led to the discovery and characterization of new microbes [86]. By contrast, repeated sampling of populations and individuals lends ecological context to infection dynamics through time. Questions regarding infection prevalence and shedding at the population scale in association with season, age cohort or reproductive phenology can be addressed, as well as long-term patterns between population demographics and infection status [87,88]. Tracking individual bats using passive integrated transponder (PIT) tags [89], tattooing [67], satellite/radio transmitters [90] or other long-term marking methods facilitates monitoring of infections or seroconversion rates. Tracking data also elucidates bat and bat-associated microbe movement between roosts (i.e. metapopulation insight) and allows estimation of population size over time. Combining host and/or parasite population genetics with infection studies also holds promise for better understanding patterns of bat dispersal and migration [91]. Future bat movement research would benefit from PIT tag data sharing (e.g. https://www.ausbats.org.au/pit-tag-register.html) to facilitate repeat detections across broad geographical areas. This would be particularly valuable for epidemiological insights into bats with long-distance migratory and dispersal behaviours.

### (c) New modelling approaches to understand viral dynamics

Multidisciplinary modelling approaches integrate theory, fieldwork and laboratory work, and allow for holistic approaches to mechanistically understand complex bat–microbe systems [66]. Empirical studies of bat infection traditionally use antibody or microbe detection in populations to construct time-series curves of active infection and exposure. While useful for hypothesis generation, integrative research is needed to identify causal drivers of dynamics, and to predict times and locations of spillover risk. Integration of age into serological time series can improve estimates of key infection parameters (e.g. R0 and force of infection) [92,93]. Age-structured serological data has been used to evaluate models of filovirus and henipavirus dynamics in Madagascar fruit bats; however, evidence of within-host variation in immunological status through time and limited model recovery of serological patterns among age classes suggests alternative dynamics may underlie viral persistence in these bat species [74]. In addition, molecular tests often contain more information than the binary presence/absence reported. As recently demonstrated with human testing data, cross-sectional viral load distributions have been used to estimate epidemic trajectories by drawing from information in cycle threshold (Ct) values from reverse transcription quantitative PCR tests [94]. This method has yet to be applied to wildlife populations and may be beneficial in cases where Ct values reflect a (probabilistic) measure of time since infection. Careful consideration of Ct values may also improve researchers' ability to determine when bats are shedding infectious viruses and estimate the risk of viral spillover [95]. Viral shedding and serology data are not regularly paired in bat–virus systems [96], though this can yield powerful insights to triangulate mechanisms of infection dynamics [97].

Sequencing and further characterization of samples positive for viral infection is necessary to understand strain diversity, identify specific molecular or phenotypic traits, and construct virus phylogenies [98]. Given the rapid evolution of viral species compared with their bat hosts, virus phylodynamics can provide insight into host movement and past transmission over the landscape [99]. Similarly, population genetic studies of bat hosts can elucidate mechanisms and pathways for present and future disease transmission [100]. Furthermore, sequencing can allow the identification of co-circulating virus strains [99]. Sequencing complete viral genomes is necessary to investigate viral recombination; incorporation of novel genes may highlight co-circulation of multiple viral families within bat populations [101]. Obtaining sequences depends on the ability to sample actively infected bats—a challenge for acute infections [88]. Phylogenetic information can thus be obtained by sampling not only the bat host but also sentinel spillover species. Sequencing viral genomes allows for the design of more inclusive molecular panels. Divergent viruses may be missed by conventional PCR [102], and while these assays are important to inform population-scale viral dynamics, they may miss nuanced virus-specific patterns in a particular bat system, especially in viral discovery efforts where *a priori* knowledge of viruses is missing.

## 4. Research at the community scale: multi-species dynamics and complexities

As with all species, bats do not exist in an ecological vacuum; thus, insights gained from individual- or population-scale studies must be re-examined within a multi-species framework. Bat infection research at the community scale involves interactions between two or more species (e.g. bats, livestock, humans) and can have great relevance to human, wildlife, agricultural and ecosystem health.

### (a) Linking host infection dynamics to spillover risk

With the large number of emerging infectious diseases reported from wildlife, often causing high morbidity and mortality, zoonotic spillover has become a great source of concern [103], and information to enable prediction and prevention of spillover is imperative. When considering spillover of microbes from wildlife to other species, there are three broad categories to consider—the reservoir species, the infectious agent and the recipient host [104]. However, these factors are not mutually exclusive and can be influenced by extrinsic variables such as climate and food availability [72,105].

Insights into bat reservoir infection dynamics and interactions with susceptible (spillover) hosts are needed to make informed risk assessments and require longitudinal research approaches [88]. Identification of spillover risk factors can be achieved by assessing infection dynamics in the reservoir host in conjunction with data on known spillover events [8]. For newly recognized viruses or those of unknown zoonotic potential, identifying possible risk factors or bridge hosts for spillover is more challenging. Closely related host species or individuals within a species may differ significantly in host proteins bound by viruses (e.g. angiotensin converting enzyme 2 (ACE2) bound by SARS-CoV-2 or dipeptidyl peptidase 4 (DPP4) bound by Middle East Respiratory Syndrome (MERS)-CoV), making predictions of susceptibility difficult [106]. In addition, a lack of expertise in bat species identification and continued changes to host and microbe taxonomy pose real challenges for standardizing analyses across temporal and spatial scales. Host–microbe datasets should specify details of bat species identification and be linked with taxonomic resources to reconcile nomenclature changes over time (e.g. https://batnames.org/). Infection dynamics can also vary across virus species and reservoir hosts, and between geographically dispersed populations of the same host species. For example, in a monoestrous *R. aegyptiacus* population in South Africa, peaks of henipavirus-related virus excretion occurred during the winter and were thought to be driven by concurrent waning of maternal immunity and nutritional stress [67]. Consequently, spillover risk was considered highest during winter and in plantations where bats were seeking food, thereby increasing the potential for human contact [67]. By contrast, *R. aegyptiacus* populations in more equatorial regions display bimodal polyoestry [107] and are subject to different climates and food availability [108], potentially altering viral excretion dynamics and the timing of peak henipavirus spillover risk.

### (b) Interfaces and behaviours promoting microbe transmission

Agricultural intensification has been linked to increased interactions and microbe spillover from bats to livestock [109,110]. For instance, the expansion of cattle farming in Latin America has allowed *D. rotundus* to feed almost exclusively on livestock blood, driving more frequent bat–livestock interactions [111,112]—a concern given their role in the transmission of rabies virus and potentially other zoonoses (e.g. *Bartonella*, *Trypanosoma*). Generally, bat–livestock interfaces are less studied than other wildlife–livestock interfaces in the context of infectious diseases [113]. Further surveillance is needed to detect spillover of bat microbes to livestock, given that asymptomatic infections may go unnoticed [114,115]. Beyond traditional microbe surveillance, movement trackers, proximity loggers and acoustic surveys can uncover patterns of overlapping landscape use [116], while surveys of farmers can provide insight into common bat–livestock interactions [117].

Urban habitats represent one interface where bats and people may come into contact. While a meta-analysis found that areas with intermediate and high levels of urban development were associated with lower bat habitat use [118], many species can adapt to human-dominated landscapes. Some bats use human infrastructure (e.g. tunnels, bridges, houses) as their roosting sites [119], sometimes sustaining large colony sizes near humans. These interactions can result in microbe transmission, such as with histoplasmosis, caused by inhaling *Histoplasma capsulatum* spores that grow on bat guano [120]. Within the flying fox (*Pteropus* spp.)–Hendra virus system, loss of native foraging habitat combined with planting of cultivated trees in urban and agricultural areas has brought bats into closer proximity with humans and horses, thereby increasing viral spillover risk [8,121]. More data are needed on the ways, frequency and duration in which humans and bats contact each other to improve estimates of spillover risk [122–124].

### (c) Anthropogenic disturbances and bat infection

Anthropogenic disturbances on bats are diverse and occur at different spatial scales and with varying severity (e.g. land modification, light and noise pollution, cave tourism, guano mining, hunting [125]). Changes in bat behaviour or community composition in response to these disturbances can alter infection and parasitism dynamics. Deforestation, through changes in bat community and roosting behaviour, has been linked to differences in the richness and prevalence of viruses and parasites across multiple Neotropical systems [79,126,127]. Anthropogenic disturbances might also cause physiological changes (e.g. stress-induced immune suppression) that increase susceptibility, reactivate latent infections [98] or increase shedding of infectious particles. Though other stressors such as food shortages, poor nutrition and fungal infection have been linked to greater viral seroprevalence, shedding and replication in bats [72,128,129], evidence for effects of direct anthropogenic disturbances on infection dynamics is limited. During periods of early and late reproduction, female Mexican free-tailed bats (*Tadarida brasiliensis*) roosting in bridges had higher rabies virus seroprevalence than those roosting in caves [130]. Other work found that *T. brasiliensis* roosting in bridges had lower plasma cortisol levels and ectoparasite loads compared with their cave-roosting counterparts [131]. Future research to resolve the effects of anthropogenic disturbance on bat infection will need to incorporate qualitative and quantitative metrics of disturbance [132,133] and assess multiple behavioural and physiological bat responses to these anthropogenic changes.

### (d) Novel approaches to reduce transmission of bat microbes

Culling of reservoir species has been employed in numerous wildlife systems to reduce disease transmission [134], yet culling outcomes in bats can be complex and may contribute to increased microbe transmission [135–137]. Reducing microbe spillover from free-ranging bats to other hosts requires a better understanding of infection dynamics from the individual to the community scales to effectively target control measures and interventions. Innovative ‘low-tech’ methods to prevent cross-species transmission between bats and other hosts, such as culturally tailored community outreach tools [7] and cost-effective physical barriers to transmission (e.g. bamboo skirts for Nipah virus [138]), should be integrated with landscape-level interventions such as ecological engineering to reduce contact with people and livestock [139] and vaccination of host species. Vaccination could complement or replace culling as proactive spillover risk reduction; however, vaccine delivery is challenging given large, reclusive bat populations. While oral vaccines held inside edible baits have been successfully implemented in some wildlife disease systems [140], the diets of most bat species preclude this approach. Novel approaches to vaccine distribution include the use of aerosolized spray vaccines [141], which are promising for cavity-roosting bat species in which large groups aggregate at high density. Alternatively, self-spreading vaccines exploit bat behaviours to spread vaccines from founder individuals to direct contacts (transferable vaccines) [142] or over multiple generations (transmissible vaccines) [143]. These methods are being investigated for combating vampire bat-transmitted rabies virus [11,144], and have potential utility in other bat–virus systems. Vaccines can also provide avenues for bat conservation (e.g. for bat populations threatened by WNS [145]).

## 5. Strengthening interdisciplinary collaboration in bat research

Historically, the bat research community has been siloed between the infectious disease and ecology/conservation disciplines, with few influential researchers bridging interdisciplinary science between these disciplines [6,146]. The emergence of WNS in the US represented one instance in which researchers came together to combat an infectious disease threatening the viability and conservation of bat populations [146]. Following the coronavirus disease (COVID)-19 pandemic, the culture of the bat research community has shifted to adopt a more integrative, interdisciplinary and collaborative approach (electronic supplementary material, figure S1). The focus on bats as sarbecovirus hosts during the pandemic had negative impacts on bats and conservation programmes [147–149] but also created an area of common concern that brought research communities together.

This momentum towards interdisciplinary collaboration in the peer-reviewed literature has been mirrored in professional networks. Global research communities joined forces to address knowledge gaps surrounding SARS-CoV-2-associated threats to bats [150,151], and facilitate regional bat One Health surveillance [152]. The International Union for Conservation of Nature (IUCN) Bat Specialist Group (https://www.iucnbsg.org/) mobilized a working group during the COVID-19 pandemic to develop guidelines for researchers, cavers, guano collectors and wildlife rehabilitators to prevent SARS-CoV-2 transmission from humans to bats, and led a zoonotic diseases science communication workshop [153]. The Global Union of Bat Diversity Networks has convened multiple networks spanning conservation to infectious diseases and initiated numerous interdisciplinary projects (https://www.gbatnet.org/interdisciplinary-projects/). The Bat Health Foundation (https://www.bathealthfoundation.org/) seeks to build a database for bat physiological parameters to inform conservation and infectious disease research. Additional interdisciplinary partnerships and projects will be critical to advance a One Health mission.

## 6. Conclusion

**Figure 2.**
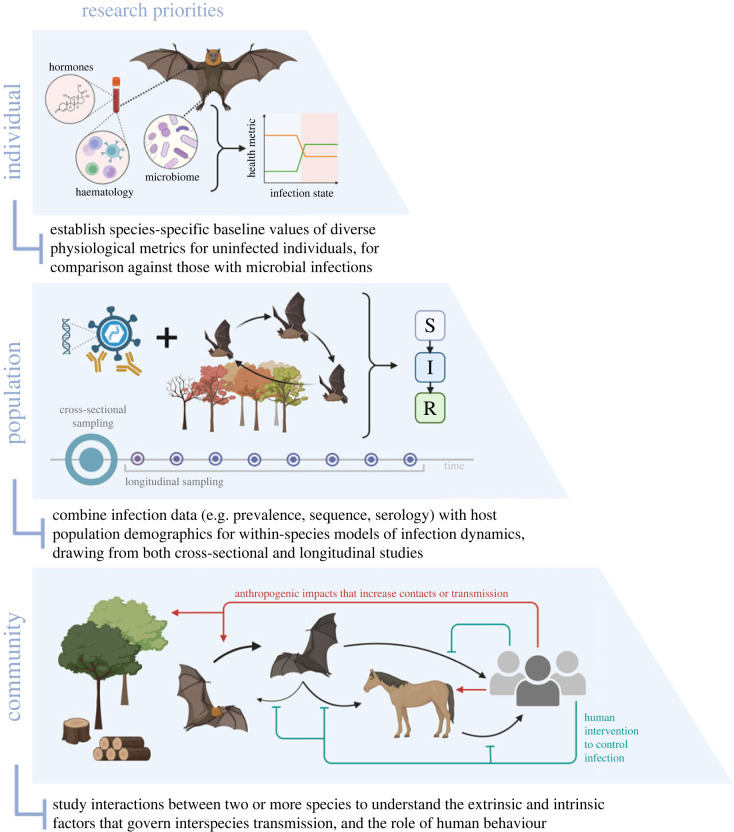
Overarching research priorities for future studies on bat infection dynamics, organized at the individual, population and community scales; S, susceptible; I, infected; R, recovered.

We have highlighted our current understanding of factors impacting bat–microbe interactions at individual, population and community scales, and identified future research needs (figure 2), including: (i) establishing species-specific baseline values for individual physiological biomarkers (especially in free-ranging populations) and including broad metrics of bat responses to infection, (ii) combining infection prevalence, sequence and serology data with host population ecology, physiology and phenology to create more informative models of infection dynamics, especially through the synthesis of cross-sectional and longitudinal studies, and (iii) generating an understanding of the extrinsic and intrinsic factors impacting microbe spread between species in communities, with special attention to the role of humans and environmental factors in these dynamics. In all cases, emphasis should be placed on communication and collaboration within the bat research community and across disciplines. Through integrated research, we can discover patterns and make predictions that will safeguard bats, humans and other species.

## Data Availability

Data to support authorship network mapping of the bat research community (described in the electronic supplementary material) are available at Zenodo: https://doi.org/10.5281/zenodo.8003910 [154]. Supplementary material is available online [155].
